# Association between periradicular lesions of posterior maxillary teeth and maxillary sinus pathologies

**DOI:** 10.1007/s10266-026-01422-3

**Published:** 2026-06-02

**Authors:** Selin Süyek, İsmail Ongun, Berk Çeli̇kkol, Oğuz Tavşan

**Affiliations:** 1 Department of Endodontics, Faculty of Dentistry, Usak University, Usak, Türkiye; 2 Department of Oral and Maxillofacial Radiology, Faculty of Dentistry, Usak University, Usak, Türkiye

**Keywords:** Maxillary sinus, Periradicular lesion, Odontogenic maxillary sinusitis, Mucosal thickening

## Abstract

This study aimed to retrospectively evaluate the association between periradicular lesions in maxillary premolar and molar roots and pathological changes in the maxillary sinus using cone-beam computed tomography (CBCT). CBCT scans including the maxillary posterior region were retrospectively analyzed. A total of 300 patients and 540 maxillary premolar and molar roots with periradicular lesions meeting the inclusion criteria were included. The presence of periradicular lesions and the proximity of the affected roots to the maxillary sinus were evaluated. Maxillary sinus pathologies were classified as mucosal thickening (MT) or odontogenic maxillary sinusitis (OMS). Associations between categorical variables were analyzed using Fisher’s exact test with Monte Carlo correction and the Pearson chi-square test. Multiple comparisons were performed using the Bonferroni-corrected Z-test. Statistical significance was set at *p* < 0.05. A significant association was found between tooth root type, including premolar and molar root subtypes, and the presence of MT and OMS (*p* < 0.001). The palatal root of the maxillary first molar showed the strongest association with maxillary sinus involvement, whereas premolar roots showed a weaker association than molars. In male patients, MT associated with single-rooted maxillary first premolars (*p* = 0.044) and OMS associated with the palatal root of the maxillary first molar (*p* = 0.003) were more frequent than in female patients. The palatal root of the maxillary first molar was the root type most strongly associated with both MT and OMS. Although premolars showed a weaker association with sinus pathology than molars, their potential role in OMS should not be overlooked.

## Introduction

The root apices of maxillary premolar and molar teeth are in close anatomical proximity to the maxillary sinus. Therefore, endodontic procedures performed on these teeth require careful consideration due to the risk of complications such as root canal perforation and the possible leakage of irrigation solutions or filling materials into the sinus cavity [1]. It has been reported that periradicular lesions in maxillary posterior teeth may induce inflammatory changes in the Schneiderian membrane even in the absence of sinus floor perforation. Furthermore, the maxillary sinus is susceptible to infections originating from the oral cavity, and odontogenic infections are considered an important etiological factor of sinusitis [2].

Evaluation of dental pathologies in the maxillary posterior region is particularly important in the diagnosis and management of maxillary sinusitis [3]. Previous studies have shown that the combined management of sinusitis associated with dental infections can lead to complete resolution of the infection and reduce the risk of recurrence [4]. Especially in persistent sinusitis cases, dental treatment such as endodontic therapy or tooth extraction may be required in addition to medical management [3].

Two-dimensional radiographic imaging methods have been reported to be insufficient for accurately determining the relationship between periradicular lesions and the maxillary sinus. Cone-beam computed tomography (CBCT) provides detailed three-dimensional visualization of the maxillary sinus and surrounding structures, allowing more accurate assessment of the relationship between periradicular lesions and the sinus membrane. For this reason, CBCT has become an important diagnostic and treatment planning tool in endodontic practice [5].

Previous studies have suggested that the palatal root of the maxillary first molar has a stronger anatomical relationship with the maxillary sinus than the roots of other maxillary posterior teeth [1, 6]. However, other teeth such as maxillary premolars and second molars may also influence the maxillary sinus and its mucosal membrane [7]. Although several studies have evaluated the relationship between maxillary posterior teeth and the maxillary sinus [8], evidence regarding the etiological role of individual tooth roots, including premolar roots, in odontogenic maxillary sinusitis remains limited. In addition, existing studies primarily focus on anatomical proximity and do not provide clinically applicable information to guide diagnostic and treatment decisions [1, 6–8]. A better understanding of root-specific associations may help clinicians more accurately assess the risk of sinus involvement and support decision-making regarding imaging and treatment.

Therefore, the aim of this study was to retrospectively evaluate the association between periradicular lesions of maxillary premolar and molar roots and sinus mucosal thickening (MT) and odontogenic maxillary sinusitis (OMS). The null hypothesis of this study was that there is no difference among maxillary posterior tooth roots with periradicular lesions in their association with odontogenic maxillary sinusitis.

## Materials and methods

### Study design and ethical approval

This study was conducted at the Departments of Endodontics and Oral and Maxillofacial Radiology, Faculty of Dentistry, Usak University. CBCT images of the maxillary posterior region obtained from patients who presented to the Faculty of Dentistry at Usak University for various reasons between 2020 and 2025 were retrospectively evaluated. Ethical approval was obtained from the Usak University Non-Interventional Clinical Research Ethics Committee (Decision No: 475-475-22).

### CBCT image acquisition

All CBCT images were acquired using a cone-beam computed tomography device (Gendex GXDP-700, KaVo Kerr, Biberach, Germany) at the Department of Oral and Maxillofacial Radiology. CBCT scans were obtained with a field of view (FOV) of 6 × 4 cm, 90 kV, 13 mA, and an exposure time of 12 s, corresponding to a small field of view covering the unilateral maxillary posterior region. The voxel size of the scans was 0.085 mm.

### Image evaluation

The images were analyzed in axial, coronal, and sagittal sections using RadiAnt DICOM Viewer software (Medixant, Poznan, Poland). The evaluations were performed by one oral and maxillofacial radiology specialist and one endodontic research assistant.

Periradicular lesions in at least one premolar or molar tooth root and the proximity of these roots to the maxillary sinus were assessed. Teeth located at a distance of ≤ 0.5 mm from the sinus floor and teeth with roots extending into the sinus cavity were recorded as teeth adjacent to the sinus [9].

### Evaluation of maxillary sinus pathologies

Maxillary sinus pathologies were identified and classified as MT and OMS.

### Mucosal thickening (MT)

**Fig. 1:**
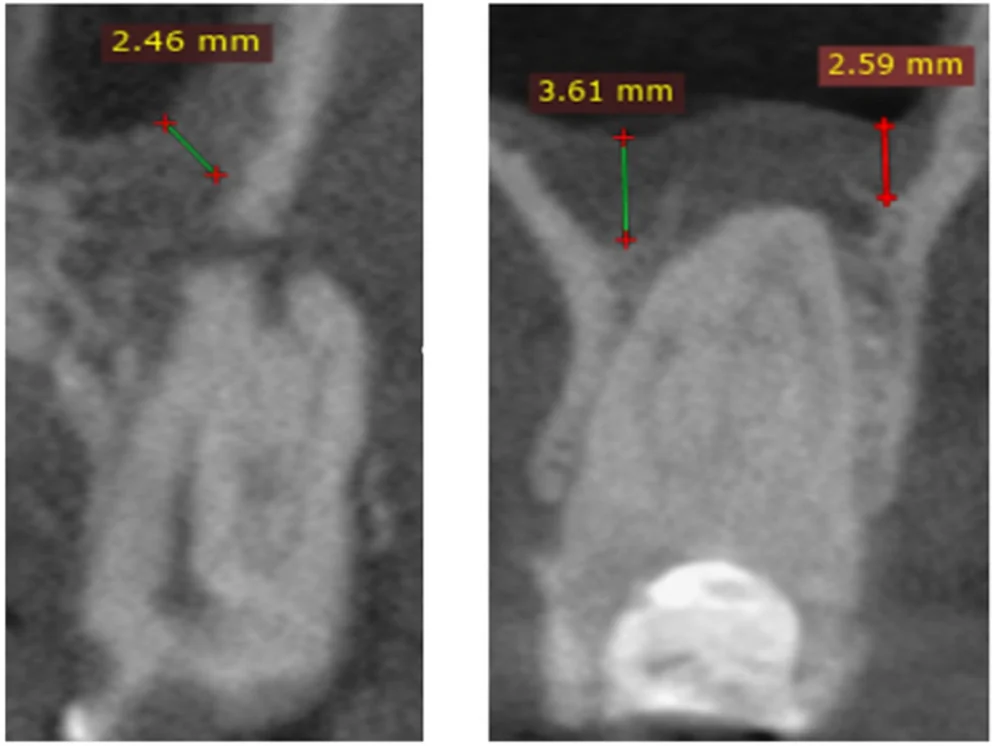
CBCT images of the roots associated with MT in the maxillary sinus: coronal section showing both roots of the maxillary second premolar and all roots of the maxillary second molar

MT was defined as an increase in the thickness of the sinus mucosa. Although different thresholds have been reported in the literature, MT of ≥ 2 mm was considered pathological in the present study [7] (Fig. 1).

### Odontogenic maxillary sinusitis (OMS)

**Fig. 2:**
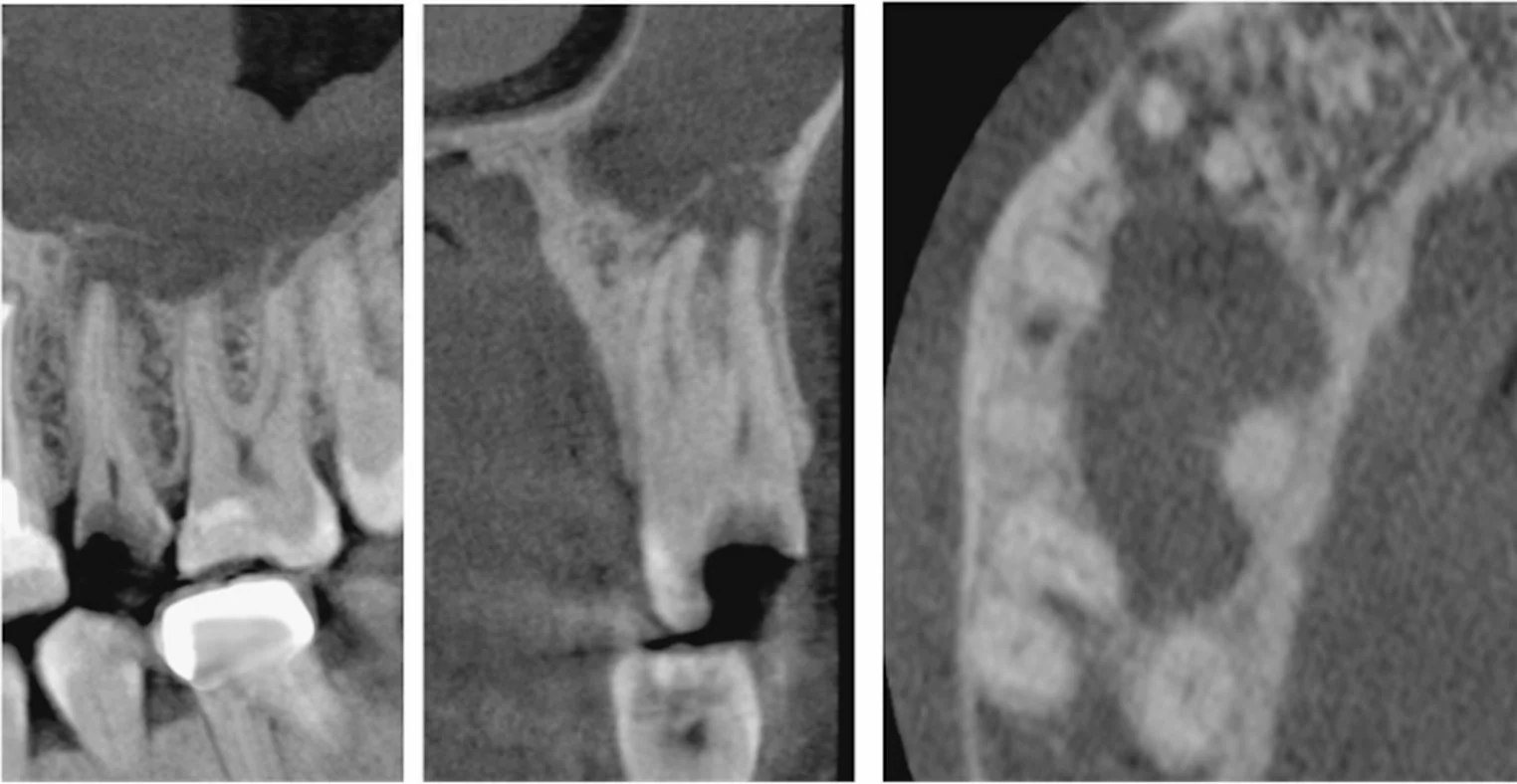
CBCT images of both roots of the maxillary second premolar associated with OMS, shown in sagittal, coronal, and axial sections

**Fig. 3:**
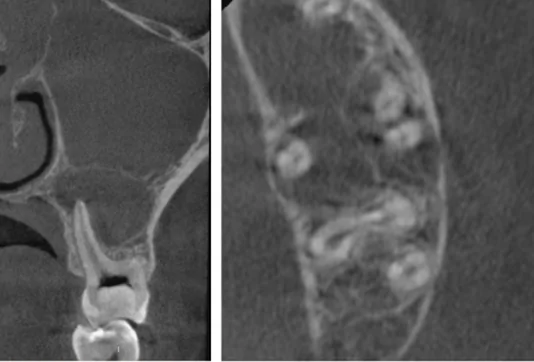
CBCT images of the palatal root of the maxillary first molar associated with OMS, shown in coronal and axial sections

OMS was diagnosed based on CBCT findings including radiolucent air bubbles within sinus opacification, decreased air content of the sinus cavity, increased radiopacity involving most or the entire sinus cavity, generalized MT of the sinus mucosa, and MT leading to ostium obstruction [10] (Figs. 2 and 3).

### Inclusion and exclusion criteria

CBCT images were included if the following criteria were met:

(1) presence of at least one maxillary first or second premolar or molar in the scanned region;

(2) presence of a periradicular lesion in the relevant tooth;

(3) proximity of the periradicular lesion to the maxillary sinus; and.

(4) absence of previous root canal treatment that could interfere with image interpretation.

CBCT images were excluded if any of the following conditions were present:

(1) absence of maxillary first or second premolar or molar teeth in the scanned region;

(2) absence of periradicular lesions, as the study specifically focused on evaluating the effects of periradicular lesions on the maxillary sinus;

(3) lesions not affecting or adjacent to the maxillary sinus;

(4) presence of previous root canal treatment in the relevant tooth; or.

(5) presence of maxillary third molars.

### Study groups

The evaluated roots were categorized into four groups according to tooth type and root morphology:

Group 1: Maxillary first premolars.

1a – buccal root.

1b – palatal root.

1c – single root.

Group 2: Maxillary second premolars.

2a – buccal root.

2b – palatal root.

2c – single root.

Group 3: Maxillary first molars.

3a – mesiobuccal root.

3b – distobuccal root.

3c – palatal root.

Group 4: Maxillary second molars.

4a – mesiobuccal root.

4b – distobuccal root.

4c – palatal root.

### Sample size calculation

The sample size was calculated using G*Power software (version 3.1). Based on the F test family (one-way ANOVA), an effect size of f = 0.2, a significance level of α = 0.03, a statistical power of 80% (1 − β), and assuming equal sample sizes among groups, the required minimum sample size was calculated as 540 roots distributed across 12 groups, with at least 45 samples per group.

### Statistical analysis

Statistical analyses were performed using R software (version 4.4.1). Associations between categorical variables were analyzed using the Pearson chi-square test or the Monte Carlo–corrected Fisher’s exact test. Comparisons of quantitative variables between groups were performed using one-way analysis of variance (ANOVA). In addition, univariate binary logistic regression analysis was performed to evaluate the effect of root type on the presence of odontogenic maxillary sinusitis and mucosal thickening. The palatal root of the maxillary first molar was used as the reference category, and odds ratios (ORs) with 95% confidence intervals (CIs) were calculated. Quantitative data were presented as mean ± standard deviation or median (minimum–maximum), while categorical data were presented as frequency and percentage. The level of statistical significance was set at *p* < 0.05.

## Results

### Patient characteristics

A total of 300 patients whose CBCT images were analyzed were included in the study. Of these, 151 (50.3%) were female and 149 (49.7%) were male. The median age was 36 years, and the mean age was 38.38 years.

### Mucosal thickening

In maxillary first premolars, MT was observed in 14.4% of buccal roots, 12.7% of palatal roots, and 5.9% of single-rooted teeth. In maxillary second premolars, the rates were 3.4% in both buccal and palatal roots and 14.4% in single-rooted teeth.

In maxillary first molars, MT was detected in 33.9% of mesiobuccal roots, 35.6% of distobuccal roots, and 39% of palatal roots. In maxillary second molars, the rates were 15.3% for mesiobuccal roots, 11% for distobuccal roots, and 12.7% for palatal roots.

A statistically significant association was found between root type and sinus MT (*p* < 0.001). The highest prevalence of MT was observed in the palatal root of the maxillary first molar (Table 1).

**Table 1:** Evaluation of the association between root type and mucosal thickening

Root	Result		Univariate		p^x^
	Not affected	Affected	OR (%95 CI)	p	
Maxillary first premolar buccal root	101 (85.6)^c^	17 (14.4)	0.263 (0.14: 0.496)	**< 0.001**	**< 0**,**001**^**x**^
Maxillary first premolar palatal root	103 (87.3)^c^	15 (12.7)	0.228 (0.118: 0.439)	**< 0.001**	
Maxillary first premolar single root	111 (94.1)^c^	7 (5.9)	0.099 (0.042: 0.231)	**< 0.001**	
Maxillary second premolar buccal root	114 (96.6)^c^	4 (3.4)	0.055 (0.019: 0.159)	**< 0.001**	
Maxillary second premolar palatal root	114 (96.6)^c^	4 (3.4)	0.055 (0.019: 0.159)	**< 0.001**	
Maxillary second premolar single root	101 (85.6)^c^	17 (14.4)	0.263 (0.14: 0.496)	**< 0.001**	
Maxillary first molar mesiobuccal root	78 (66.1)^ab^	40 (33.9)	0.803 (0.472: 1.365)	0.417	
Maxillary first molar distobuccal root	76 (64.4)^a^	42 (35.6)	0.865 (0.51: 1.467)	0.590	
Maxillary first molar palatal root	72 (61)^a^	46 (39)	Reference		
Maxillary second molar mesiobuccal root	100 (84.7)^bc^	18 (15.3)	0.282 (0.151: 0.526)	**< 0.001**	
Maxillary second molar distobuccal root	105 (89)^c^	13 (11)	0.194 (0.098: 0.384)	**< 0.001**	
Maxillary second molar palatal root	103 (87.3)^c^	15 (12.7)	0.228 (0.118: 0.439)	**< 0.001**	

^x^Monte Carlo–corrected Fisher’s Exact Test; Frequency (percentage);

^a–c^ Proportions sharing the same letter are not significantly different.; OR (95% CI): Odds ratio (95% confidence interval); Bold values indicate statistically significant differences

Univariate binary logistic regression analysis showed that, compared with the palatal root of the maxillary first molar, all premolar and maxillary second molar roots had significantly lower odds of mucosal thickening (*p* < 0.001), whereas no significant differences were observed for the distobuccal (*p* = 0.590) and mesiobuccal (*p* = 0.417) roots of the maxillary first molar (Table 1).

In the gender-based evaluation, sinus mucosal thickening in the single-rooted group of the maxillary first premolar was observed at a rate of 11.3% in males and 1.5% in females, and this difference was found to be statistically significant (*p* = 0.044). Sinus mucosal thickening rates in the buccal and palatal roots of the maxillary first premolar, in all root types of the maxillary second premolar, and in the root groups of the maxillary first and second molars were similar between males and females, and no significant association with gender was found (*p* > 0.05; Table 2).

**Table 2:** Association between gender and root types for mucosal thickening and odontogenic maxillary sinusitis

Root	Mucosal Thickening			Odontogenic maxillary sinusitis		
	Male (%)	Female (%)	*p*	Male (%)	Female (%)	*p*
Maxillary first premolar buccal root	6 (11.3)	11 (16.9)	0.550ᵇ	5 (5.2)	4 (4.7)	0.569^a^
Maxillary first premolar palatal root	5 (9.4)	10 (15.4)	0.492ᵇ	7 (7.3)	3 (3.5)	0.338^a^
Maxillary first premolar single root	6 (11.3)	1 (1.5)	**0.044ᵃ**	5 (5.2)	2 (2.3)	0.449^a^
Maxillary second premolar buccal root	2 (3.8)	2 (3.1)	1.000ᵃ	7 (7.3)	4 (4.7)	0.664ᵇ
Maxillary second premolar palatal root	2 (3.8)	2 (3.1)	1.000ᵃ	9 (9.4)	5 (5.8)	0.534ᵇ
Maxillary second premolar single root	9 (17.0)	8 (12.3)	0.649ᵇ	15 (15.6)	15 (17.4)	0.897ᵇ
Maxillary first molar mesiobuccal root	16 (30.2)	24 (36.9)	0.567ᵇ	44 (45.8)	30 (34.9)	0.133^c^
Maxillary first molar distobuccal root	19 (35.8)	23 (35.4)	1.000ᵇ	42 (43.8)	36 (41.9)	0.797^c^
Maxillary first molar palatal root	23 (43.4)	23 (35.4)	0.485ᵇ	59 (61.5)	34 (39.5)	**0.003** ^**c**^
Maxillary second molar mesiobuccal root	6 (11.3)	12 (18.5)	0.415ᵇ	33 (34.4)	33 (38.4)	0.576^c^
Maxillary second molar distobuccal root	5 (9.4)	8 (12.3)	0.841ᵇ	31 (32.3)	30 (34.9)	0.712^c^
Maxillary second molar palatal root	7 (13.2)	8 (12.3)	1.000ᵇ	28 (29.2)	30 (34.9)	0.409^c^

^a^ Fisher’s Exact Test;

^b^ Yates correction

^c^ Pearson’s chi-square test. Values are presented as frequency (percentage). Bold values indicate statistically significant differences

### Odontogenic maxillary sinusitis

In maxillary first premolars, OMS was observed in 5% of buccal roots, 5.5% of palatal roots, and 3.9% of single-rooted teeth. In maxillary second premolars, the rates were 6% in buccal roots, 7.7% in palatal roots, and 16.5% in single-rooted teeth.

In maxillary first molars, the rates of OMS were 40.7% for mesiobuccal roots, 42.9% for distobuccal roots, and 51.1% for palatal roots. In maxillary second molars, the rates were 36.3%, 33.5%, and 31.9% for mesiobuccal, distobuccal, and palatal roots, respectively.

A statistically significant association was found between root type and OMS (*p* < 0.001). The highest prevalence of OMS was observed in the palatal root of the maxillary first molar (Table 3).

**Table 3:** Evaluation of the association between root type and odontogenic maxillary sinusitis

Root	Result		Univariate		p^x^
	Not affected	Affected	OR (%95 CI)	p	
Maxillary first premolar buccal root	173 (95)^c^	9 (5)	0.05 (0.024: 0.103)	**< 0.001**	**< 0**,**001**^**x**^
Maxillary first premolar palatal root	172 (94.5)^cd^	10 (5.5)	0.056 (0.028: 0.112)	**< 0.001**	
Maxillary first premolar single root	175 (96.1)^c^	7 (3.9)	0.038 (0.017: 0.086)	**< 0.001**	
Maxillary second premolar buccal root	171 (94)^cd^	11 (6)	0.062 (0.031: 0.121)	**< 0.001**	
Maxillary second premolar palatal root	168 (92.3)^cd^	14 (7.7)	0.08 (0.043: 0.148)	**< 0.001**	
Maxillary second premolar single root	152 (83.5)^d^	30 (16.5)	0.189 (0.116: 0.308)	**< 0.001**	
Maxillary first molar mesiobuccal root	108 (59.3)^ab^	74 (40.7)	0.656 (0.433: 0.993)	**0.046**	
Maxillary first molar distobuccal root	104 (57.1)^ab^	78 (42.9)	0.718 (0.475: 1.085)	0.116	
Maxillary first molar palatal root	89 (48.9)^b^	93 (51.1)	Reference		
Maxillary second molar mesiobuccal root	116 (63.7)^ab^	66 (36.3)	0.544 (0.358: 0.828)	**0.004**	
Maxillary second molar distobuccal root	121 (66.5)^a^	61 (33.5)	0.482 (0.316: 0.737)	**0.001**	
Maxillary second molar palatal root	124 (68.1)^a^	58 (31.9)	0.448 (0.292: 0.686)	**< 0.001**	

^x^Pearson chi-square test; Frequency (percentage);

^a–d^ Proportions sharing the same letter are not significantly different; Test statistic: 347.546; OR (95% CI): Odds ratio (95% confidence interval); Bold values indicate statistically significant differences

Univariate binary logistic regression analysis showed that, compared with the palatal root of the maxillary first molar, all premolar (*p* < 0.001) and maxillary second molar roots (*p* ≤ 0.004) had significantly lower odds of OMS. No significant difference was observed for the distobuccal root (*p* = 0.116), whereas a significant difference was observed for the mesiobuccal root (*p* = 0.046) of the maxillary first molar (Table 3).

In the gender-based evaluation, a statistically significant difference was observed only in the palatal root of the maxillary first molar, with higher OMS rates in males 61.5% compared to females 39.5% (*p* = 0.003). No significant gender-related differences were found in the root types of the maxillary first and second premolars, the mesiobuccal and distobuccal roots of the maxillary first molar, or any root groups of the maxillary second molar (*p* > 0.05; Table 2).

## Discussion

The roots of maxillary premolar and molar teeth are in close anatomical proximity to the maxillary sinus. It has been reported that periradicular lesions developing in these teeth may induce inflammatory changes in the Schneiderian membrane even in the absence of sinus floor perforation. Because the maxillary sinus is susceptible to infections originating from the oral cavity, odontogenic infections are considered important etiological factors of sinusitis [1, 2]. In the present study, the effects of periradicular lesions in maxillary posterior tooth roots on sinus MT and OMS were retrospectively evaluated using CBCT.

Accurate evaluation of the maxillary sinus largely depends on the imaging method used. Although panoramic radiography is widely used in clinical practice, it has several limitations, including geometric distortion and inadequate visualization of structures such as the mesial wall of the maxillary sinus, which may reduce its reliability in detecting sinus pathologies [11]. In contrast, CBCT provides detailed three-dimensional visualization of the maxillary sinus and allows accurate assessment of the relationship between periradicular lesions and the sinus membrane. Additionally, CBCT offers advantages such as relatively low radiation dose and high spatial resolution, making it a valuable imaging modality in endodontic diagnosis and treatment planning [12]. For these reasons, CBCT imaging was used in the present study.

In the present study, the prevalence of OMS was found to be 60.7%. Although this rate is higher than the prevalence values reported in earlier studies, which ranged between 10% and 30% [13, 14], it is consistent with more recent CBCT-based studies indicating that more than half of maxillary sinusitis cases may be of odontogenic origin [2, 15, 16]. The use of three-dimensional imaging methods such as CBCT may contribute to improved detection and increased diagnostic sensitivity for odontogenic sinusitis.

A statistically significant association was found between tooth roots and both MT and OMS (*p* < 0.001). Among the evaluated roots, the palatal root of the maxillary first molar showed the strongest association with sinus pathology. In contrast, premolar roots demonstrated a weaker association compared with molar roots. The findings indicate that premolar and second molar roots have significantly lower odds of OMS compared to the palatal root of the maxillary first molar, whereas the distobuccal root shows no significant difference and the mesiobuccal root shows only a modest reduction. This suggests that the increased risk is primarily associated with the maxillary first molar region, with the palatal root representing the highest risk site. Based on these findings, the null hypothesis of the study was rejected. The results obtained are consistent with previous studies reporting that molar teeth, particularly the maxillary first molar, play a more prominent role in the pathogenesis of OMS [17].

Previous studies have reported that the maxillary first molar is one of the teeth located closest to the maxillary sinus floor, and that the palatal root of this tooth often shows the strongest anatomical relationship with the sinus [18, 19]. However, some studies have also indicated that in certain populations the roots of the maxillary second molars may be located at an equal or even closer distance to the sinus floor [8, 20]. These anatomical relationships may contribute to the development of OMS in the presence of periradicular lesions and should therefore be carefully considered during clinical evaluation.

Another possible explanation for the prominent role of the maxillary first molar is its early eruption and prolonged exposure to the oral environment. Because these teeth erupt earlier than other posterior teeth, they may be more frequently exposed to irritants such as dental caries and trauma. When this situation is combined with the close anatomical proximity of the palatal root of the first molar to the maxillary sinus floor, inflammatory changes in the sinus mucosa and the development of OMS may occur in the presence of periradicular lesions [21, 22]. However, current evidence does not indicate that the longer presence of a tooth in the oral cavity alone is a direct etiological factor for OMS.

Although maxillary premolar teeth are generally located farther from the sinus floor than molars, previous studies have reported that the roots of maxillary second premolars may occasionally be positioned in close proximity to the sinus floor [23]. Such proximity may increase the likelihood of MT and the development of OMS [24, 25]. Similarly, in the present study, premolar roots showed a weaker association with OMS compared with molar roots. However, within the premolar groups, the single-rooted maxillary second premolar, as well as the buccal root and single root of the maxillary first premolar, were more frequently associated with OMS. These findings are supported by a CBCT-based population study reporting that approximately 94% of maxillary second premolars are single-rooted and that the distance between the root apex and the sinus floor may be considerably reduced in these teeth. In contrast, in two-rooted premolars the roots often diverge laterally, which may decrease their proximity to the sinus floor [26].

No significant differences were observed among the remaining premolar root groups. Therefore, although premolar teeth generally represent a lower-risk group compared with molars, their potential contribution to OMS should not be overlooked in the presence of periradicular lesions.

MT/OMS was observed more frequently in male patients in certain root groups. The higher prevalence of MT/OMS in male patients may be associated with behavioral factors such as smoking and oral hygiene habits, both of which have been linked to increased sinus mucosal changes and odontogenic infections [27]. In addition, it has been reported that males may have a larger maxillary sinus volume and a reduced distance between posterior tooth roots and the sinus floor [28], which may further contribute to this increased susceptibility.

Several studies investigating the relationship between maxillary sinus MT and odontogenic infections have reported that pathologies originating from posterior teeth located in different anatomical positions may similarly lead to MT. Retrospective studies have also shown that no significant association exists between the distance of a periradicular lesion to the sinus floor and MT [29]. In contrast, other studies have suggested that the severity of apical periodontitis may be associated with increased MT [4]. In the present study, similar effects on the sinus mucosa were observed when periradicular lesions were present in more than one tooth. Therefore, the evaluation focused primarily on the presence of sinusitis rather than on measuring MT in millimeters.

The integration of CBCT into routine clinical practice plays an important role in the evaluation of odontogenic pathologies in the maxillary posterior region [30, 31]. CBCT enables three-dimensional assessment of the relationship between the sinus floor and tooth roots, the position of root apices, and possible perforation risks that cannot be reliably detected using two-dimensional imaging methods [32]. Although periapical radiographs may often be sufficient for premolar teeth, CBCT provides higher diagnostic accuracy, particularly for maxillary molars located close to the sinus floor [17]. Consequently, CBCT imaging may contribute to improved diagnostic accuracy, prevention of unnecessary surgical interventions, and optimization of endodontic treatment planning according to anatomical variations [33].

Although the distance between the root apices and the maxillary sinus provides valuable anatomical information, it should be interpreted as a surrogate marker rather than a definitive determinant of treatment planning. These measurements alone are insufficient to dictate clinical decisions and must be considered together with clinical findings, patient symptoms, and other radiographic features. CBCT-based distance assessment may assist clinicians in estimating the potential risk of sinus involvement and in planning endodontic or surgical interventions; however, it should not be used as a standalone criterion. In addition, CBCT-based measurements are subject to certain limitations, including voxel size, image artifacts, and inter-observer variability, which may influence measurement accuracy. Therefore, these factors should be taken into account when interpreting the results.

The main limitation of this study is its retrospective design. Because of the retrospective nature of the study, potential confounding factors such as patients’ previous history of sinusitis, systemic diseases, and sinonasal pathologies could not be controlled. In addition, the diagnosis was based solely on radiological findings, and clinical symptoms could not be evaluated. The inclusion of only teeth with periradicular lesions may limit the generalizability of the findings; however, this approach was necessary to address the specific research objective. Although age data were available, it was not included in the statistical analysis. Furthermore, other confounding factors such as smoking status and history of non-odontogenic sinusitis could not be assessed. These limitations should be considered when interpreting the results. Future prospective multicenter studies including clinical, systemic, and demographic variables are needed to further clarify the relationship between odontogenic sinusitis and non-odontogenic sinusitis.

The detection of sinus mucosal changes associated with maxillary posterior tooth roots presenting periradicular lesions suggests that dental infections may directly influence sinus health. These findings highlight the importance of considering both the affected tooth and its anatomical relationship with the maxillary sinus during endodontic treatment planning. In this context, CBCT plays an important role in evaluating the distance between the root apex and the sinus floor, the thickness of the Schneiderian membrane, and potential perforation risks.

CBCT imaging may be particularly recommended in cases with suspected odontogenic involvement [34], especially when conventional imaging is insufficient or symptoms persist. In particular, periradicular lesions in maxillary molars may warrant earlier use of CBCT due to their higher likelihood of sinus involvement. Furthermore, maxillary molar roots, particularly the palatal root of the maxillary first molar, should be prioritized during evaluation due to their stronger association with sinus pathology.

In patients with periradicular lesions and findings of OMS, endodontic treatment should be considered the primary approach. A multidisciplinary approach, including an ENT specialist, may be beneficial in cases with sinus involvement or persistent symptoms.

## Conclusion

The palatal root of the maxillary first molar showed the strongest association with sinus MT and OMS. Although maxillary premolar teeth demonstrated a weaker association with sinus pathology compared with molars, their potential role in the development of OMS should not be overlooked. Given that OMS constitutes a considerable proportion of sinusitis cases, dental etiology should be considered, particularly in patients with treatment-resistant or recurrent chronic sinusitis. In such cases, the presence of periradicular lesions in maxillary posterior teeth should be carefully evaluated during treatment planning. Clinicians should prioritize the evaluation of maxillary molar roots, particularly the palatal root of the maxillary first molar. Referral to an ENT specialist should be considered in cases with extensive sinus pathology or persistent symptoms despite appropriate dental treatment.

## Data Availability

The datasets generated and/or analyzed during the current study are not publicly available but are available from the corresponding author upon reasonable request.
